# Effects of Exercise on Autonomic Cardiovascular Function in Older Adults: A Systematic Review and Meta-Analysis

**DOI:** 10.1007/s40279-025-02357-5

**Published:** 2025-11-20

**Authors:** Paula Etayo-Urtasun, Mikel Izquierdo, Mikel L. Sáez de Asteasu

**Affiliations:** 1 Department of Health Sciences, Navarrabiomed, Hospital Universitario de Navarra (HUN), Universidad Pública de Navarra (UPNA), IdiSNA, 31008 Pamplona, Spain; 2 CIBER of Frailty and Healthy Aging (CIBERFES), Instituto de Salud Carlos III, Madrid, Spain

## Abstract

**Background:**

Physical exercise has been proposed to enhance cardiovascular autonomic function; however, current evidence in older populations remains controversial.

**Objective:**

This systematic review and meta-analysis aimed to examine the effects of physical exercise on autonomic cardiovascular function in older adults.

**Methods:**

A systematic literature search was conducted in PubMed, Web of Science, Scopus, and ScienceDirect on March 12, 2025, following PRISMA 2020 guidelines. Two independent reviewers applied the PICOS model to screen randomised controlled trials (RCTs) published since 2010 that investigated the effects of exercise interventions on autonomic cardiovascular function in older adults. Methodological quality was assessed using the PEDro scale. Standardised mean differences (SMD) and 95% confidence intervals (CI) were calculated through random effects models using the Empirical Bayes method. This systematic review and meta-analysis was registered in PROSPERO (CRD420250651364).

**Results:**

Fifteen RCTs were included in the meta-analysis. Exercise interventions significantly increased the root mean square of the successive differences (RMSSD) (SMD 0.636, 95% confidence interval [CI] 0.014–1.258; *p* = 0.045) and significantly decreased the low-frequency / high-frequency (LF/HF) ratio (SMD − 0.506, 95% CI − 0.954 to − 0.057; *p* = 0.027). No significant effects were found for the standard deviation of normal-to-normal intervals (SDNN) (SMD 0.718, 95% CI − 0.120 to 1.557; *p* = 0.093) or baroreflex sensitivity (SMD − 0.137, 95% CI − 0.670 to 0.396; *p* = 0.614). Although substantial heterogeneity was noted, no evidence of publication bias was observed.

**Conclusion:**

These results highlight the utility of structured exercise as a nonpharmacological tool to improve autonomic cardiovascular function in older adults, with potential implications for reducing cardiovascular risk and promoting healthy ageing.

**Supplementary Information:**

The online version contains supplementary material available at 10.1007/s40279-025-02357-5.

## Key Points

**Table:** 

Exercise improves key markers of parasympathetic activity (RMSSD, pNN50, LF/HF), supporting better autonomic balance in older adults.
Programs with aerobic or progressive-load exercise lasting ≥12 weeks show the strongest benefits for heart rate variability in ageing populations.
This rigorous meta-analysis confirms that older adults can still improve autonomic function through exercise, reinforcing its clinical value.

## Introduction

The autonomic nervous system (ANS) is essential for maintaining physiological homeostasis through the regulation of involuntary functions such as heart rate, blood pressure, digestion, and respiratory function [1]. Structurally and functionally, the ANS consists of two primary divisions, the sympathetic and parasympathetic nervous systems, which generally exhibit complementary roles [2, 3]. Specifically, the sympathetic nervous system facilitates adaptive responses during threatening or stressful situations, whereas the parasympathetic nervous system predominates in restful and restorative states [4, 5].

Ageing is frequently characterised by a progressive decline in cardiac vagal modulation, primarily due to diminished parasympathetic activity and enhanced sympathetic tone [6, 7]. These alterations contribute to an imbalance in the ANS branch that regulates cardiac function and vascular tone [2, 7]. Autonomic cardiovascular dysfunction emerges as a prevalent and complex geriatric syndrome that is significantly linked to frailty, impaired functional capacity, and disability [8]. Importantly, diminished autonomic modulation of cardiac function is a substantial predictor of adverse cardiovascular outcomes and increased all-cause mortality [6, 9, 10].

Autonomic cardiovascular function can be examined by employing various methodologies, including heart rate variability (HRV), baroreflex sensitivity (BRS), heart rate recovery (HRR), and blood pressure variability (BPV) [4]. Among these, HRV is the most frequently utilised owing to its non-invasive nature and robust prognostic capability, where reduced HRV has been consistently linked to a higher risk of falls and increased mortality in older populations [4, 7, 11]. HRV analysis quantifies the fluctuations between successive electrocardiogram R-R intervals and autonomic regulation of the heart [4, 12]. HRV metrics encompass time-domain, frequency-domain, and nonlinear analyses. Key time-domain indices include the standard deviation of normal-to-normal intervals (SDNN), the root mean square of successive differences (RMSSD), and the percentage of successive NN intervals differing by more than 50 ms (pNN50) [10, 13, 14]. Frequency domain analysis evaluates low-frequency power (LF), high-frequency power (HF), and the LF/HF ratio, which provides specific insights into sympathetic and parasympathetic modulation [13, 14].

Additionally, nonlinear approaches, such as Poincaré plot analysis (SD1 and SD2) and entropy-based methods, further elucidate the complexity and adaptability of heart rate dynamics [15]. Furthermore, the BRS measures autonomic function and evaluates autonomic capability to regulate blood pressure via the baroreceptor reflex [4]. Similarly, reduced HRR post-exercise is recognised as an independent marker of impaired autonomic function and has been associated with elevated cardiovascular and all-cause mortality risks [16]. BPV further enhances the assessment of autonomic function, as greater BPV is correlated with increased cardiovascular morbidity and mortality [17].

Although ageing is generally associated with autonomic dysfunction, emerging evidence emphasises that such deterioration is not solely age-dependent, but also influenced by lifestyle factors [12]. Observational studies have indicated that regular physical activity correlates with favourable HRV indices in older adults, suggesting protective effects against autonomic decline [18].

While randomised controlled trials (RCT) have convincingly demonstrated improvements in autonomic cardiovascular function following exercise interventions in middle-aged adults, evidence among older populations remains limited and inconsistent [19, 20]. Previous systematic reviews have acknowledged these discrepancies, but the absence of quantitative analyses has prevented conclusive determination of the magnitude of exercise-related effects [15]. Similarly, Raffin et al. [13] conducted a meta-analysis that included a limited number of studies, restricting the generalisability and robustness of their conclusions.

Therefore, these methodological gaps underscore the necessity for a comprehensive synthesis of the existing literature on the influence of physical exercise on autonomic cardiovascular function in older adults. Given the prognostic relevance of autonomic dysfunction, particularly in frail or clinical populations, current position papers have emphasised the role of structured exercise in maintaining autonomic integrity and cardiovascular resilience in older adults [21]. Consequently, this systematic review and meta-analysis aimed to evaluate the impact of physical exercise interventions on autonomic cardiovascular function, thereby providing a clearer understanding of exercise-induced adaptations in this growing population.

## Methods

### Study Design

This meta-analysis followed the guidelines of the Preferred Reporting Items for Systematic Reviews and Meta-Analyses (PRISMA) Statement [22]. It was registered in PROSPERO (CRD420250651364).

### Search Strategy

The literature search was performed on March 12, 2025. Articles were gathered from Medline (PubMed), Web of Science, Scopus, and ScienceDirect databases. Two authors (PE-U and MLSA) searched independently, and any discrepancies were resolved by consulting a third author (MI). A methodological search filter was established to include studies published from 2010 onwards, with no language restrictions applied. The detailed search strategy was as follows: (exercise[Title] OR "physical activity"[Title] OR training[Title]) AND (autonomic[Title/Abstract] OR "heart rate variability"[Title/Abstract] OR "baroreflex sensitivity"[Title/Abstract] OR “heart rate recovery”[Title/Abstract] OR “blood pressure variability”[Title/Abstract]) AND (older OR senior* OR aged OR geriatric OR ageing OR aging).

### Selection Criteria

Inclusion criteria were established using the PICOS model (Population, Intervention, Comparison, Outcome, Study type) [23]. Thus, articles were included if they met the following criteria: (a) included older adults (aged ≥ 60 years) who were not physically active; (b) evaluated the impact of an exercise intervention involving whole-body aerobic, strength, or power exercises; (c) included a group following usual lifestyle or a stretching programme; (d) measured cardiovascular autonomic function by HRV, BRS, HRR, or BPV; and (e) were RCTs. Reviews, conference abstracts, and editorials were not included. Studies in which the control group performed a structured exercise protocol were also excluded.

### Screening

The search strategy involved pooling results from the four databases and eliminating duplicates. Two reviewers (PE-U and MLSA) screened the articles by their titles and abstracts. Subsequently, articles were read and selected based on the established inclusion criteria.

### Data Extraction

Two authors (PE-U and MLSA) independently extracted the following key data from each study: general information (e.g., first author's name and year), participants (e.g., number of subjects and age), intervention (e.g., duration and type of exercise), measurement methods (e.g., device, time, position, and sample rate), and outcomes (HRV, BRS, HRR, or BPV). HRV outcomes were categorised into time-domain indices (SDNN, RMSSD, and pNN50), frequency-domain indices (LF, HF, and LF/HF), and non-linear indices (SD1 and SD2). The meta-analysis included studies that measured time-domain indices in milliseconds, frequency-domain indices in normalised units, and nonlinear indices in milliseconds.

### Risk of Bias Assessment

Two authors (PE-U and MLSA) independently evaluated the risk of bias in each study using the Physiotherapy Evidence Database (PEDro) scale, a robust 11-item scale designed to assess the methodological quality of RCTs [24]. The evaluation was displayed in a graphical format using Review Manager (RevMan) 5.3.

### Statistical Analysis

A meta-analysis was performed on the outcomes analysed in at least three studies. We assessed heterogeneity across RCTs using Cochran’s *Q* test and the Higgins *I*^2^ statistic [25, 26]. Due to the heterogeneity among studies, we applied a random effects model using the Empirical Bayes method to calculate the standardised mean difference (SMD) and 95% confidence interval (CI) [27]. Selection models estimated the publication bias [28]. Sensitivity analyses were conducted by applying Pearson’s *r* values of 0.2 and 0.8. In cases where a study contributed disproportionately to the overall variance, sensitivity analyses were carried out by excluding the outlying study. The significance level was set at *p* < 0.05. Statistical analyses were performed using JASP software, version 0.18.3 (2024).

## Results

### Study Selection

**Fig. 1:**
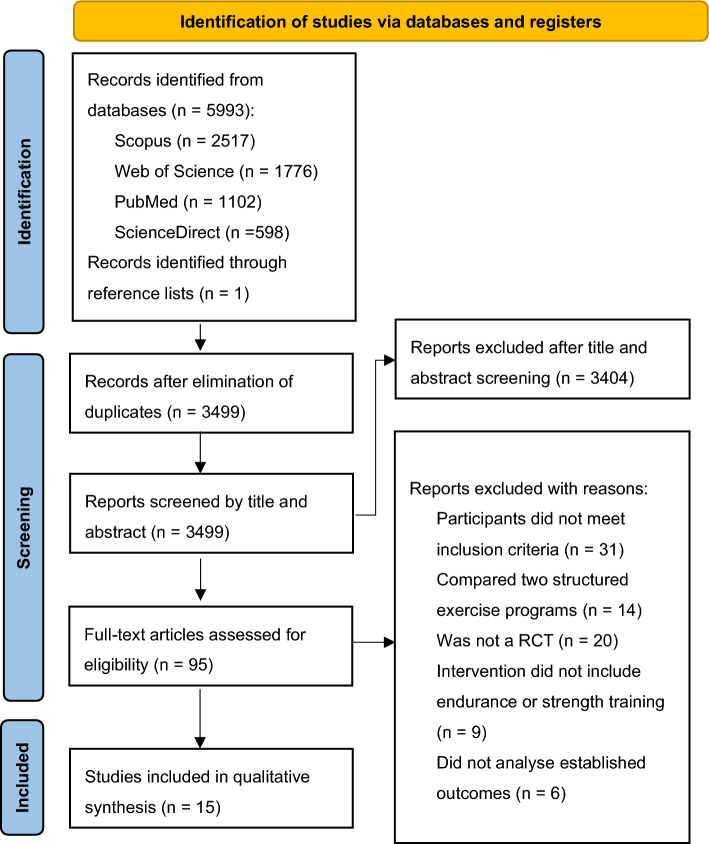
Flow diagram of the database search process. *RCT* randomised controlled trial

Figure 1 illustrates a flowchart summarising the literature search and the selection process. Initially, 5993 studies were identified through searches of four databases: Scopus (*n* = 2517), Web of Science (*n* = 1776), PubMed (*n* = 1102), and Science Direct (*n* = 598). An additional study was identified from the reference lists, yielding 5994 records. After removing 2495 duplicates, 3499 articles underwent title and abstract screening. Ninety-five full-text articles were evaluated for eligibility, 80 of which were excluded because they did not meet the inclusion criteria. Ultimately, 15 articles were included in the qualitative synthesis, and 10 were eligible for quantitative meta-analysis.

### Risk of Bias Assessment

The risk of bias assessment for the included studies is summarised in online Figs. S1 and S2 in the electronic supplementary material (ESM). Overall, the methodological quality ranged from moderate to high, with most studies adequately addressing essential bias domains.

### Participant Characteristics

Table 1 summarises the characteristics of the participants from the 15 included studies, comprising 540 older adults (191 men and 349 women) [19, 20, 29–41]. Most studies enrolled healthy community-dwelling older adults. However, several studies specifically included individuals with chronic conditions, such as coronary artery disease [35], heart failure [36], hypertension [37, 38], and depression [39].

**Table 1:** Qualitative analysis of studies evaluating the effects of exercise interventions on autonomic cardiovascular function

Study	Patient characteristics	Population type	Intervention characteristics	Measurement methods	Significant changes in IG compared to CG
Albinet et al. [29]	N (M/F): 24 (11/13) Mean age ± SD: 70.7 ± 4.2 IG/CG: 12/12	Not disease-specific	IG: 12-week aerobic exercise programme CG: stretching programme	Polar RS 800, 5-min seated at rest	↑SDNN (ms), ↑RMSSD (ms), ↔ LF (ms^2^/Hz), ↑HF (ms^2^/Hz)
Albinet et al. [30]	N (M/F): 36 (10/26) Mean age ± SD: 66.5 ± 5 IG/CG: 19/17	Not disease-specific	IG: 21-week aquarobics and swimming training CG: stretching programme	Polar RS 800, 5-min seated at rest	↔ RMSSD (ms), ↑Ln RMSSD (ms), ↔ LF (ms^2^/Hz), ↔ Ln LF (ms^2^/Hz), ↔ HF (ms^2^/Hz), ↑Ln HF (ms^2^/Hz), ↑HF/(LF + HF)
Buto et al. [31]	N (M/F): 21 (6/15) Mean age ± SD: 75.7 ± 6.6 IG/CG: 12/9	Not disease-specific	IG: 16-week aerobic, strength and balance exercises CG: usual lifestyle	ECG, 15-min supine position at rest, 1000 Hz	↔ BRS (ms/mmHg)
Costa Chaves et al. [32]	N (M/F): 30 (26/4) Mean age ± SD: 69.3 ± 6.6 IG/CG: 15/15	Not disease-specific	IG: 8-week power exercises and walking CG: usual lifestyle	ECG, 20-min supine position at rest, 600 Hz	↔ SDNN (ms), ↔ RMSSD (ms), ↔ LF (nu), ↔ LH (nu)
Gambassi et al. [19]	N (M/F): 26 (0/26) Mean age ± SD: 65 ± 3 IG/CG: 13/13	Not disease-specific	IG: 12-week resistance training CG: usual lifestyle	ECG, 20-min supine position at rest, 600 Hz	↑SDNN (ms), ↑RMSSD (ms), ↑pNN50 (%), ↔ LF (ms^2^), ↑HF (ms^2^), ↓LF (nu), ↑HF (nu), ↓LF/HF (nu)
Gerage et al. [20]	N (M/F): 29 (0/29) Mean age ± SD: 65.9 ± 4.6 IG/CG: 15/14	Not disease-specific	IG: 12-week resistance training CG: stretching programme	Polar s810i, 10-min seated position at rest	↔ SDNN (ms), ↔ RMSSD (ms), ↔ LF (ms^2^), ↔ LF (nu), ↔ HF (ms^2^), ↔ HF (nu), ↔ LF/HF (nu), ↔ SD1 (ms), ↔ SD2 (ms)
Kanegusuku et al. [33]	N (M/F): 25 (7/18) Mean age ± SD: 63.5 ± 4 IG/CG: 12/13	Not disease-specific	IG: 16-week resistance training CG: usual lifestyle	ECG, 10-min seated position at rest	↔ LF (nu), ↔ HF (nu), ↔ LF/HF (nu), ↔ BRS (ms/mmHg)
Ksela et al. [34]	N (M/F): 34 (23/11) Median age (IQR): 66 (63–70) IG/CG: 20/14	Not disease-specific	IG1: 2-week endurance exercises and calisthenics IG2: 2-week water-based endurance exercises and calisthenics CG: lifestyle advice	ECG, 20-min supine position at rest	↔ SD1, ↔ SD2
Mameletzi et al. [35]	N (M/F): 20 (20/0) Mean age ± SD: 69.6 ± 6.7 IG/CG: 10/10	Cardiovascular disease	IG: 7-month aerobic training CG: usual lifestyle	Task Force Monitor 3040i device, supine position at rest	↑BRS, ↑BEI
Murad et al. [36]	N (M/F): 66 (24/42) Mean age ± SD: 69.1 ± 5.2 IG/CG: 31/35	Cardiovascular disease	IG: 16-week walking and cycling training CG: follow-up calls	ECG, 10-min supine position at rest	↑SDNN, ↑RMSSD
Oliveira-Dantas et al. [37]	N (M/F): 25 (0/25) Mean age ± SD: 66.1 ± 5.2 IG/CG: 13/12	Hypertension	IG: 10-week resistance training CG: usual lifestyle	ECG, 10-min supine position at rest	↔ LF (ms^−2^), ↔ HF (ms^−2^), ↔ LF/HF (ms^−2^)
Sardeli et al. [38]	N (M/F): 46 (15/31) Mean age ± SD: 65.3 ± 4.4 IG/CG: 23/23	Hypertension	IG: 16-week aerobic and strength training CG: usual lifestyle	Heart rate monitor, supine position at rest	↔ SDNN (ms), ↔ RMSSD (ms), ↔ pNN50 (%), ↔ HF (ms^2^), ↔ LF (ms^2^), ↔ LF/HF (ms^2^), ↔ HF (nu), ↔ LF (nu), ↔ SD1, ↑SD2
Toni et al. [39]	N (M/F): 88 (25/63) Mean age ± SD: 74.2 ± 6.7 IG/CG: 38/50	Depression	IG: 24-week aerobic or strength exercise CG: usual lifestyle	ECG, 20-min supine position at rest	↑ pNN50 (%), ↑RMSSD (ms), ↑SDNN (ms), ↑LF (ms^2^/Hz), ↑HF (ms^2^/Hz), ↓LF/HF
Varas-Diaz et al. [40]	N (M/F): 20 (13/7) Mean age ± SD: 68.6 ± 5.7 IG/CG: 10/10	Not disease-specific	IG: 6-week exergaming-based dance training CG: 1 h education session	Polar RS800CX, supine position at rest	↑ Ln RMSSD (ms), ↔ pNN50 (%), ↑HF (nu), ↔ LF/HF (nu)
Wanderley et al. [41]	N (M/F): 50 (11/39) Mean age ± SD: 68 ± 5.5 IG1/IG2/CG: 20/11/19	Not disease-specific	IG1: 8-month aerobic training IG2: 8-month resistance training CG: usual lifestyle	Polar recorder, 5-min supine position at rest	↔ SDRR (ms), ↔ HF (ms^2^)

↑ indicates significant increase, ↓ indicates significant decrease, ↔ indicates no significant changes

*BEI* baroreflex effectiveness index, *BRS* baroreflex sensitivity, *CG* control group, *ECG* electrocardiogram, *HF* high-frequency power, *Hz* hertz, *IG* intervention group, *IQR* interquartile range, *LF* low-frequency power, *ms* milliseconds, *N(M/F)* number of participants (male/female), *nu* normalised units, *pNN50* percentage of successive NN intervals differing by more than 50 ms, *RMSSD* root mean square of successive differences, *SD* standard deviation, *SDNN* standard deviation of normal-to-normal intervals

### Intervention Characteristics

All included exercise interventions were supervised and encompassed various exercise modalities, including aerobic [29, 30, 35, 36, 40], multicomponent [31, 32, 34, 38], and resistance training programmes [19, 20, 33, 37]. Three studies employed two distinct intervention groups alongside a control group, comparing aerobic versus resistance, or water-based versus land-based exercises [34, 39, 41]. Intervention durations typically ranged from 12 to 16 weeks, with an exercise frequency between two and three sessions per week. Moderate-intensity protocols with progressive workload increases were frequently employed [19, 35, 41]. Control groups generally maintained their usual activity levels, with some receiving general lifestyle recommendations [34, 40] or participating in stretching programmes [20, 29, 30]. Additionally, one study reported sertraline administration in both intervention and control groups [39].

### Heart Rate Variability Data

**Fig. 2:**
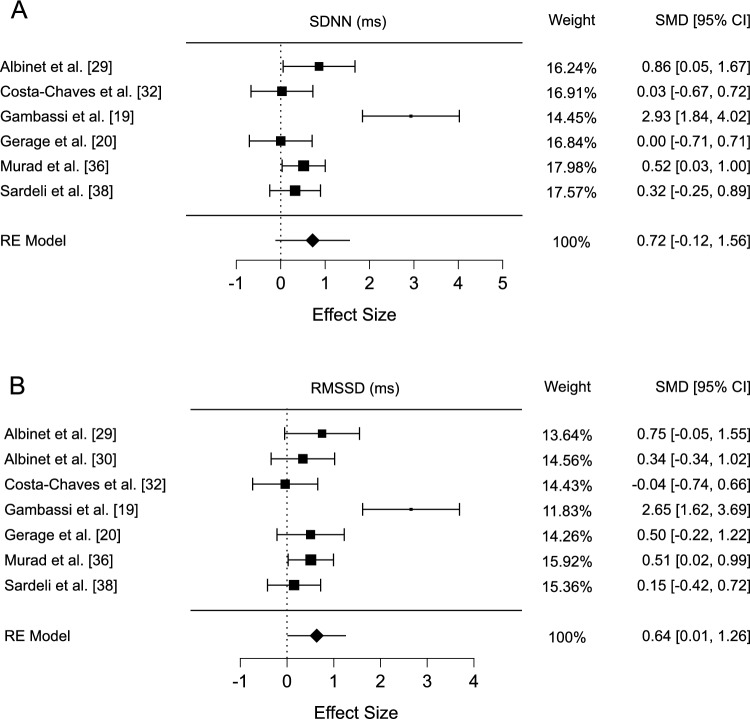
**A** Forest plot of exercise effects on SDNN, **B** forest plot of exercise effects on RMSSD. *SDNN* standard deviation of normal-to-normal intervals, *RMSSD* root mean square of successive differences, *SMD* standardised mean difference, *RE* random effects

Six studies analysed the effects of exercise on SDNN. Significant heterogeneity was observed (*Q* = 23.93, *I*^2^ = 88.9%; *p* < 0.001). No evidence of publication bias was observed (*p* = 0.568). The meta-analysis indicated no significant change in SDNN (SMD 0.718, 95% CI − 0.120 to 1.557; *p* = 0.093; Fig. 2A), which persisted after sensitivity analysis (*r* = 0.2, SMD 0.582, 95% CI − 0.084 to 0.249; *p* = 0.087; *r* = 0.8, SMD 1.042, 95% CI − 0.242 to 2.326; *p* = 0.112). However, after excluding the study by Gambassi et al. [19]—which exhibited the largest effect size but also contributed substantial variance—the increase in SDNN reached statistical significance (*Q* = 3.76, SMD 0.353, 95% CI 0.074–0.632; *p* = 0.013).

Seven studies assessed RMSSD, exhibiting significant heterogeneity (*Q* = 20.93, *I*^2^ = 82.88%; *p* = 0.002) but no publication bias (*p* = 0.716). The analysis revealed significant improvements in RMSSD following the exercise intervention (SMD 0.636, 95% CI 0.014–1.258; *p* = 0.045, Fig. 2B), which was partially confirmed by sensitivity analyses (*r* = 0.2, SMD 0.539, 95% CI 0.034–1.045; *p* = 0.037; *r* = 0.8, SMD 0.838, 95% CI − 0.053 to 1.729; *p* = 0.065). After excluding the study by Gambassi et al. [19], which showed the greatest effect size yet introduced high heterogeneity, the increase in RMSSD remained significant (*Q* = 3.21, SMD 0.360, 95% CI 0.102–0.618; *p* = 0.006).

Three studies measured pNN50 and demonstrated low heterogeneity (*Q* = 2.17, *I*^2^ = 7.64%; *p* = 0.338) and no publication bias (*p* = 0.322). The results demonstrated significant increases in pNN50 (SMD 0.642, 95% CI 0.119–1.165; *p* = 0.016, Fig. S3, see ESM), confirmed by sensitivity analyses (*r* = 0.2, SMD 0.576, 95% CI 0.077–1.075; *p* = 0.024; *r* = 0.8, SMD 0.790, 95% CI 0.088–1.491; *p* = 0.027).

**Fig. 3:**
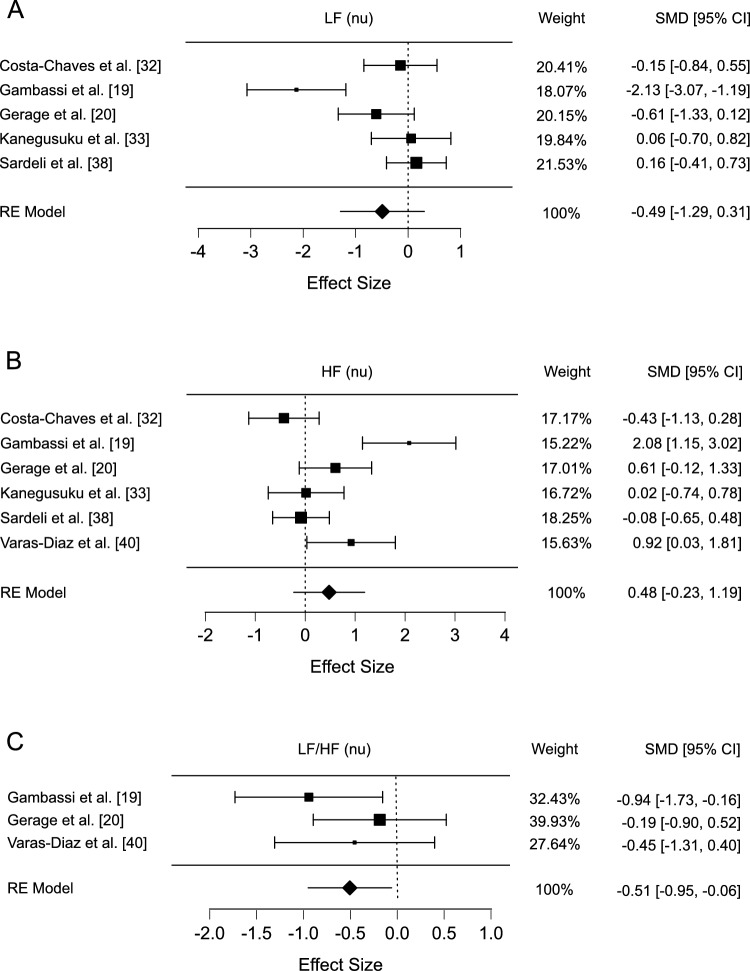
**A** Forest plot of exercise effects on LF, **B** forest plot of exercise effects on HF, **C** forest plot of exercise effects on LF/HF. *LF* low-frequency, *HF* high-frequency, *SMD* standardised mean difference, *RE* random effects

Five studies measured LF power, showing substantial heterogeneity (*Q* = 18.65, *I*^2^ = 83.56%; *p* < 0.001) without publication bias (*p* = 0.386). No significant change was observed in LF power (SMD − 0.491, 95% CI − 1.289 to 0.306; *p* = 0.227, Fig. 3A), consistent across sensitivity analyses (*r* = 0.2, SMD − 0.397, 95% CI − 1.051 to 0.256; *p* = 0.233;* r* = 0.8, SMD − 0.702, 95% CI − 1.815 to 0.410; *p* = 0.216). After excluding the study by Gambassi et al. [19], the change in LF remained statistically non-significant (*Q* = 2.85, SMD − 0.099, 95% CI − 0.633 to 0.435; *p* = 0.598).

Six studies measured HF power and reported high heterogeneity (*Q* = 22.85, *I*^2^ = 81.65%; *p* < 0.001) but without evidence of publication bias (*p* = 0.664). No significant effect was noted for HF power (SMD 0.478, 95% CI − 0.233 to 1.189; *p* = 0.188, Fig. 3B), corroborated by sensitivity analyses (*r* = 0.2, SMD 0.412, 95% CI − 0.187 to 1.010; *p* = 0.178; *r* = 0.8, SMD 0.624, 95% CI − 0.343 to 1.592; *p* = 0.206). Following the exclusion of the study by Gambassi et al. [19], the change in HF remained non-significant (*Q* = 7.72, SMD 0.163, 95% CI − 0.489 to 0.815; *p* = 0.525).

Three studies measured the LF/HF ratio. No significant heterogeneity was found among these studies (*Q* = 1.97, *I*^2^ = 0%; *p* = 0.373) and no publication bias was detected (*p* = 0.983). The meta-analysis demonstrated a significant reduction in LF/HF following exercise interventions (SMD − 0.506, 95% CI − 0.954 to − 0.057; *p* = 0.027, Fig. 3C), a finding consistent with the sensitivity analyses (*r* = 0.2, SMD − 0.461, 95% CI − 0.908 to − 0.015; *p* = 0.043; *r* = 0.8, SMD − 0.590, 95% CI − 1.070 to − 0.111; *p* = 0.016).

Three studies assessed the effect of exercise on SD1. No significant heterogeneity was observed (*Q* = 1.24, *I*^2^ = 0%; *p* = 0.538) and publication bias was not evident (*p* = 0.368). The analysis indicated no significant changes in SD1 (SMD 0.162, 95% CI − 0.209 to 0.533; *p* = 0.392, Fig. S4, see ESM), a result consistent in sensitivity analysis (*r* = 0.2, SMD 0.138, 95% CI − 0.232 to 0.508; *p* = 0.465; *r* = 0.8, SMD 0.208, 95% CI − 0.164 to 0.580; *p* = 0.273).

Additionally, three studies evaluated SD2. No heterogeneity was noted among the studies (*Q* = 0.09, *I*^2^ = 0%; *p* = 0.955) and no publication bias was identified (*p* = 0.099). Meta-analysis revealed a significant increase in SD2 after exercise intervention (SMD 0.575, 95% CI 0.198–0.951; *p* = 0.003, Fig. S5, see ESM), which was confirmed by sensitivity analyses (*r* = 0.2, SMD 0.466, 95% CI 0.092–0.840; *p* = 0.015; *r* = 0.8, SMD 0.840, 95% CI 0.454–1.226; *p* < 0.001).

### Baroreflex Sensitivity

Three studies evaluated the BRS changes resulting from exercise training. No significant heterogeneity was observed across the studies (*Q* = 2.54, *I*^2^ = 21.80%; *p* = 0.281), and there was no indication of publication bias (*p* = 0.831). Meta-analysis did not show significant effects of exercise interventions on BRS (SMD − 0.137, 95% CI − 0.670 to 0.396; *p* = 0.614, Fig. S6, see ESM), confirmed by sensitivity analyses (*r* = 0.2, SMD − 0.118, 95% CI − 0.587 to 0.352; *p* = 0.623; *r* = 0.8, SMD − 0.177, 95% CI − 0.900 to 0.545; *p* = 0.631).

A summary of the sensitivity analyses is presented in Table S1 in the ESM.

### Blood Pressure Variability and Heart Rate Recovery

Only one study assessed BPV, specifically analysing low-frequency systolic (LF_SBP_) and diastolic blood pressure (LF_DBP_) variability [33]. Although the intervention group displayed higher absolute values than the control group both pre- and post-intervention, no statistically significant changes were observed within groups from baseline to post-intervention assessments. In addition, none of the studies analysed HRR.

## Discussion

The primary finding of this meta-analysis is that physical exercise benefits several parameters of autonomic cardiovascular regulation in older adults. Our results showed significant increases in RMSSD and pNN50, suggesting improvements in short-term parasympathetic modulation and enhanced heart rate adaptability. Additionally, the observed significant reduction in the LF/HF ratio indicated a favourable shift toward increased parasympathetic dominance, while the significant increase in the nonlinear parameter SD2 suggested improved long-term autonomic adaptability. Conversely, no significant exercise-induced changes were observed in SDNN, LF, HF, SD1, and BRS. Owing to the limited availability of data, the effect of exercise on BPV and HRR remains unclear.

Improvements in RMSSD and SD2 reflect enhanced parasympathetic regulation and long-term autonomic adaptability, respectively—both of which have been associated with lower cardiovascular morbidity and all-cause mortality in ageing populations [9, 10]. Therefore, the observed changes, although moderate in magnitude, may carry clinically meaningful prognostic implications.

Contrary to the meta-analysis by Raffin et al. [13], our analysis did not detect significant improvements in SDNN among older adults following exercise interventions. However, the increase in SDNN became significant after eliminating the study by Gambassi et al. [19], which showed the largest effect size but also contributed to high variance. Additionally, in contrast to Raffin et al. [13], who reported no significant change in RMSSD, our findings align with those of other meta-analyses demonstrating significant RMSSD improvements in cardiac patients [42–44]. Regarding frequency-domain indices, our findings support those reported by Pearson and Smart [44] indicating a significant reduction in the LF/HF ratio; however, they diverge from those of dos Santos Disessa et al. [42], who noted significant increases. Our findings offer a foundation for refining clinical exercise prescriptions by identifying specific HRV indices that are more responsive to training stimuli in older adults. This paves the way for developing targeted interventions based on individual autonomic profiles.

Although BPV and HRR are well-established markers of autonomic regulation, particularly in older and clinical populations, they were infrequently reported across eligible RCTs. This absence reflects a persistent gap in exercise trials involving older adults. It underscores the need for standardised autonomic outcome reporting that includes both short-term (e.g., RMSSD, SDNN) and dynamic post-exercise measures.

Several factors may have contributed to the heterogeneity observed across the included studies, particularly variations in participant characteristics. Previous evidence indicates that autonomic cardiovascular adaptations may differ between healthy populations and individuals with chronic diseases [45]. Given the female predominance in the included studies, potential sex-specific responses to exercise interventions should be considered. Previous research suggests that hormonal status and baseline vagal tone may influence HRV responsiveness [46]. Future trials should stratify outcomes by sex to inform more individualised exercise guidelines.

Differences in the exercise protocols also represent a potential source of variability. Most studies that implemented aerobic exercise interventions found significant improvements in HRV parameters, revealing that aerobic exercise is crucial to achieve adaptations in the autonomic cardiovascular system [29, 30, 35, 36]. Regarding strength training, Grässler et al. [15] previously concluded that resistance training alone did not significantly enhance autonomic cardiac function in older populations. Nevertheless, certain resistance training protocols included in our analysis produced notable effect sizes, challenging the assertion that resistance exercises cannot improve sympathovagal balance [19].

Previous evidence has emphasised that interventions of longer duration and greater frequency yield superior HRV outcomes [13]. Our findings partially support this claim, as interventions exceeding 12 weeks frequently led to significant autonomic adaptation. However, Gambassi et al. [19] demonstrated substantial HRV improvements after a relatively shorter 12-week intervention consisting of two-weekly sessions compared with longer or more frequent exercise programmes [20, 33]. The initial sedentary status of the participants might partly explain these outcomes, as the most substantial adaptations generally occur within the first weeks of initiating regular exercise, as reported by Toni et al. [39].

Exercise intensity is a critical determinant of autonomic adaptation [47]. Most of the interventions in our analysis employed moderate-intensity protocols. Progressive increases in exercise load have significantly improved cardiac autonomic modulation in multiple studies [19, 29, 30, 35, 36, 39, 40]. Rodrigues et al. [48] specifically compared periodised and non-periodised training in older women, highlighting the superior autonomic benefits of periodised protocols. Thus, exercise prescriptions should consider optimal intensity and progression to maximise autonomic cardiovascular function, promoting beneficial adaptations without inducing excessive physiological stress [1].

The control groups across the studies varied in their intervention protocols. Most studies advised participants to maintain their usual lifestyle habits, although several employed supervised stretching routines [29, 30]. Such stretching interventions might influence autonomic measures, potentially leading to underestimation of the effects of exercise [20].

Although several plausible pathways have been proposed, the physiological mechanisms underlying exercise-induced autonomic adaptation remain unclear. Exercise may enhance cardiac vagal modulation partly by reducing angiotensin II expression [12]. Additionally, exercise-induced improvements in endothelial function and nitric oxide bioavailability contribute to enhanced vascular function, indirectly benefiting cardiac autonomic regulation [21]. Exercise also exerts anti-inflammatory and antioxidant effects, mitigating chronic inflammation and oxidative stress, both of which are associated with autonomic dysfunction [12]. Furthermore, reduction in fat mass associated with regular exercise has been suggested as a potential mechanism for enhancing parasympathetic activity [49]. Collectively, these mechanisms underscore the multifaceted role of physical exercise as a valuable nonpharmacological strategy to optimise autonomic cardiovascular health in older adults [50, 51].

**Fig. 4:**
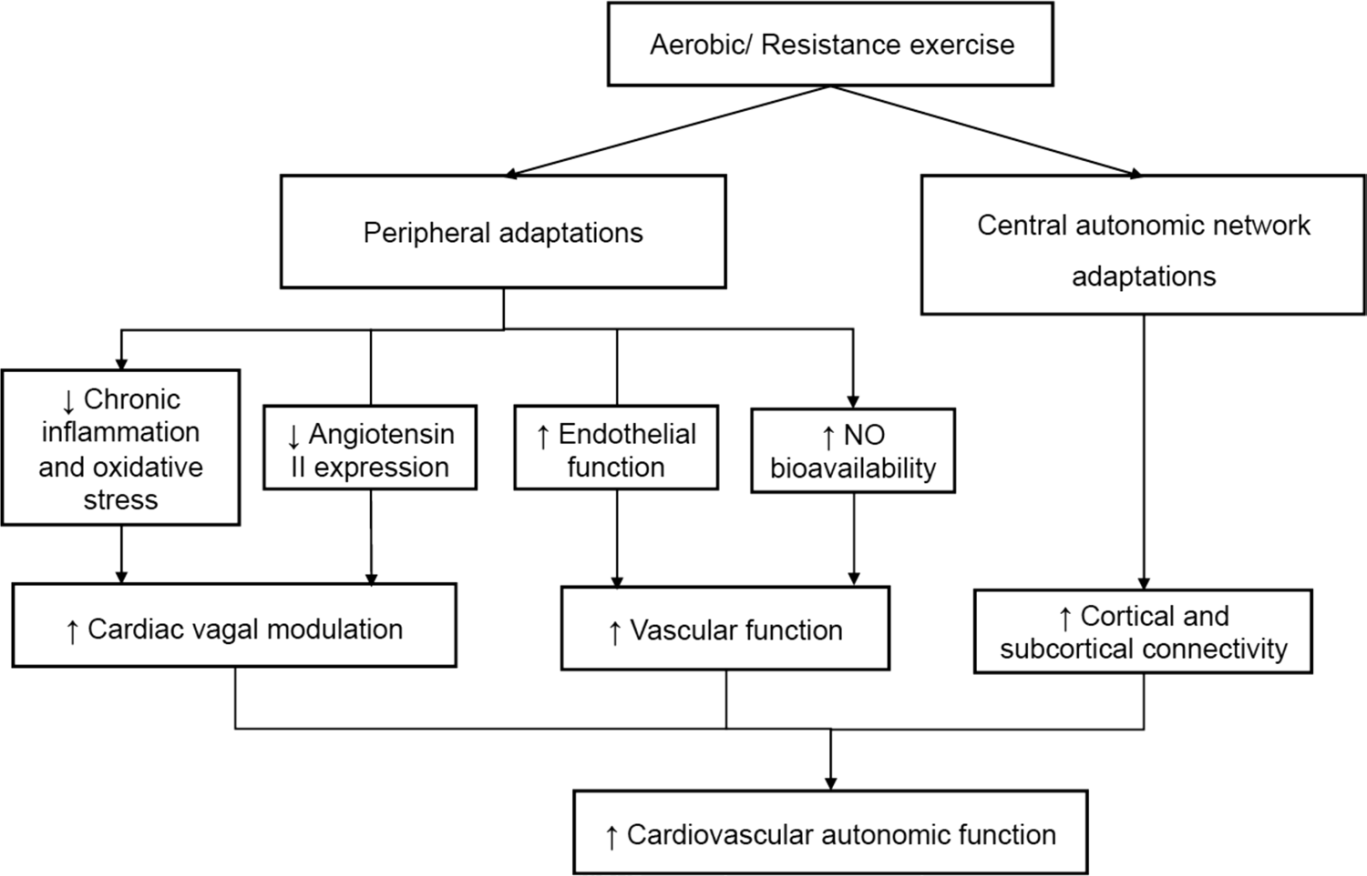
Mechanisms linking exercise and autonomic cardiovascular adaptation in older adults. *NO* nitric oxide

Beyond peripheral mechanisms, emerging evidence highlights the role of central autonomic network adaptations in mediating exercise-induced improvements in autonomic regulation [52]. Exercise may enhance cortical and subcortical connectivity, particularly involving regions such as the insula, anterior cingulate cortex, and brainstem nuclei, which are pivotal in autonomic control [53]. These central adaptations may underpin the observed changes in HRV and sympathovagal balance (Fig. 4).

The results of this meta-analysis should be interpreted in the context of several limitations. First, the sample size was relatively small, potentially limiting the statistical power to detect any significant differences. Although subgroup analyses by exercise modality or population characteristics could elucidate sources of heterogeneity, the limited number of studies per subgroup constrained our ability to perform such analyses robustly. Second, several studies exhibited methodological shortcomings, notably the lack of adequate blinding procedures, which may have introduced bias. Furthermore, the outcomes utilised in the included studies are indirect measures of autonomic cardiovascular function, potentially lacking precise sensitivity or specificity to directly quantify sympathetic or parasympathetic activity. It is noteworthy that exercise-induced changes observed in HRV might reflect adaptations in sinus node function or cardiac structural remodelling, rather than direct alterations in autonomic nervous system activity [54]. Additionally, older adults may exhibit atypical heart rate patterns associated with an increased mortality risk, potentially elevating certain HRV indices. Therefore, higher HRV values in this population may not always indicate an improved autonomic function [55]. Moreover, the studies included in this review primarily focused on short-term HRV measures. Although these measures have been validated, long-term HRV recordings (24-h HRV) may provide more accurate data, increasing reliability in autonomic assessments [14]. Finally, the limited inclusion of BPV and HRR measures in the literature restricts a more comprehensive understanding of autonomic responsiveness. Future trials should incorporate these variables using standardised methodologies to capture a broader autonomic profile.

These limitations highlight the need for future research to investigate the effects of exercise interventions on HRV in older populations. Additional studies with larger sample sizes and long-term HRV measurements are required. Further research is essential to elucidate the physiological mechanisms underlying exercise-induced enhancements in cardiac vagal modulation. Future investigations should incorporate not only traditional time-domain and frequency-domain indices, but also emphasise nonlinear HRV indices, which may offer complementary insights. Additionally, longitudinal follow-up studies could provide critical information regarding the long-term sustainability of exercise-induced autonomic improvement.

Nevertheless, our meta-analysis has numerous methodological strengths that should be considered. A major strength is the systematic, rigorous, and comprehensive approach applied to the data search, selection process, and synthesis, ensuring high methodological quality. Robust statistical methodologies, including heterogeneity assessments, sensitivity analyses, and the Empirical Bayes method, enhance the reliability and interpretability of findings. Collectively, our findings provide valuable evidence regarding the beneficial effects of exercise on sympathetic-parasympathetic balance in older adults, suggesting that ageing does not fully diminish the capacity to enhance HRV through appropriately prescribed exercise interventions.

## Conclusions

This review highlights the capacity of physical exercise to elicit favourable changes in autonomic regulation in older adults, reinforcing its role as a cornerstone of preventive geriatric care. Future trials should prioritise long-term follow-up, standardised HRV protocols, and exploration of central mechanisms to fully leverage exercise as autonomic therapy.

## Supplementary Information

Below is the link to the electronic supplementary material.

Supplementary file1 (PDF 239 KB)

## Declarations

### Data availability statement

Data corresponding to this meta-analysis will be shared upon reasonable request to the corresponding author.
