# Supplementary material for: Effects of Exercise on Autonomic Cardiovascular Function in Older Adults: A Systematic Review and Meta-Analysis
Source: Sports Med. 2025 Nov 20;56(2):507–19. doi: 10.1007/s40279-025-02357-5 (PMC12982300; doi:10.1007/s40279-025-02357-5)

Effects of exercise on cardiovascular autonomic function in older adults: a systematic review and meta-analysis

Running head: Effects of exercise on cardiovascular autonomic function in older adults

Paula Etayo-Urtasun,[1] Mikel Izquierdo,[1,2*] Mikel L. Sáez de Asteasu[1,2]

[1] Navarrabiomed, Hospital Universitario de Navarra (HUN), Universidad Pública de Navarra (UPNA), IdiSNA, Pamplona (Spain).

[2] CIBER of Frailty and Healthy Aging (CIBERFES), Instituto de Salud Carlos III, Madrid, Spain.

***Corresponding author:** Mikel Izquierdo, PhD, Department of Health Sciences, Public University of Navarre, 31008 Pamplona, Spain (mikel.izquierdo@unavarra.es).

Table of Contents

Table S1. Summary of sensitivity analyses……………………………………………….2

Figure S1. Risk of bias in each trial……………………………………………………..3

Figure S2. Risk of bias graph…………………………………………………..……....4

Figure S3. Forest plot of PNN50…………………………………..……………………4

Figure S4. Forest plot of SD1…………………………………………..………………….4

Figure S5. Forest plot of SD2……………………………………………..………………5

Figure S6. Forest plot of baroreflex sensitivity (BRS)……………………..…………….5

Table S1. Summary of sensitivity analyses.

Outcome	Main SMD (95% CI)	Sensitivity (r = 0.2)	Sensitivity (r = 0.8)	Outlier removed
SDNN (ms)	0.72 (-0.12, 1.56)	0.58 (-0.08, 0.25)	1.04 (-0.24, 2.33)	0.35 (0.07, 0.63)
RMSSD (ms)	0.64 (0.01, 1.26)	0.54 (0.03, 1.05)	0.84 (-0.05, 1.73)	0.36 (0.1, 0.62)
LF (nu)	-0.49 (-1.29, 0.31)	-0.4 (-1.05, 0.26)	-0.7 (-1.82, 0.41)	-0.1 (-0.63, 0.44)
HF (nu)	0.48 (-0.23, 1.19)	0.41 (-0.19, 1.01)	0.62 (-0.34, 1.59)	0.16 (-0.49, 0.82)
LF/HF (nu)	-0.51 (-0.95, -0.06)	-0.46 (-0.91, -0.02)	-0.59 (-1.07, - 0.11)	N/A
PNN50	0.64 (0.12, 1.17)	0.58 (0.08, 1.08)	0.79 (0.09, 1.49)	N/A
SD1	0.16 (-0.21, 0.53)	0.14 (-0.23, 0.51)	0.21 (-0.16, 0.58)	N/A
SD2	0.58 (0.2, 0.95)	0.47 (0.09, 0.84)	0.84 (0.45, 1.23)	N/A
BRS	-0.14 (-0.67, 0.4)	-0.12 (-0.59, 0.35)	-0.18 (-0.9, 0.55)	N/A

N/A: analysis not performed due to the limited number of studies.

Figure S1. Risk of bias in each trial.

	Eligibility criteria were specified	Subjects were randomly allocated to groups	Allocation was concealed	Groups were similar at baseline	All subjects were blinded	All therapists were blinded	All evaluators were blinded	Measures of at least 85% of the subjects	All subjects received treatment or control (or intention to treat analysis)	Between-group statistical comparisons	Provided point measures and measures of variability
Albinet et al. [29]	+	+	+	+	−	−	−	+	+	+	+
Albinet et al. [30]	+	+	+	+	−	−	−	+	+	+	+
Buto et al. [31]	+	+	+	−	−	−	+	−	+	+	+
Costa Chaves et al. [32]	+	+	+	+	−	−	−	+	+	+	+
Gambassi et al. [19]	+	+	+	+	−	−	−	+	+	+	+
Gerage et al. [20]	+	+	+	+	−	−	+	+	+	+	+
Kanegusuku et al. [33]	+	+	+	+	−	−	−	−	−	+	+
Ksela et al. [34]	+	+	+	+	−	−	−	+	+	+	+
Mameletzi et al. [35]	+	+	+	+	−	−	+	−	+	+	+
Murad et al. [36]	+	+	+	+	−	−	+	−	+	+	+
Oliveira-Dantas et al. [37]	+	+	+	+	−	−	+	+	+	+	+
Sardeli et al. [38]	+	+	+	+	−	−	−	+	+	+	+
Toni et al. [39]	+	+	+	−	−	−	+	+	+	+	+
Varas-Díaz et al. [40]	+	+	+	+	−	−	−	+	+	+	+
Wanderley et al. [41]	+	+	+	−	−	−	−	−	+	+	+

Figure S2. Risk of bias graph.

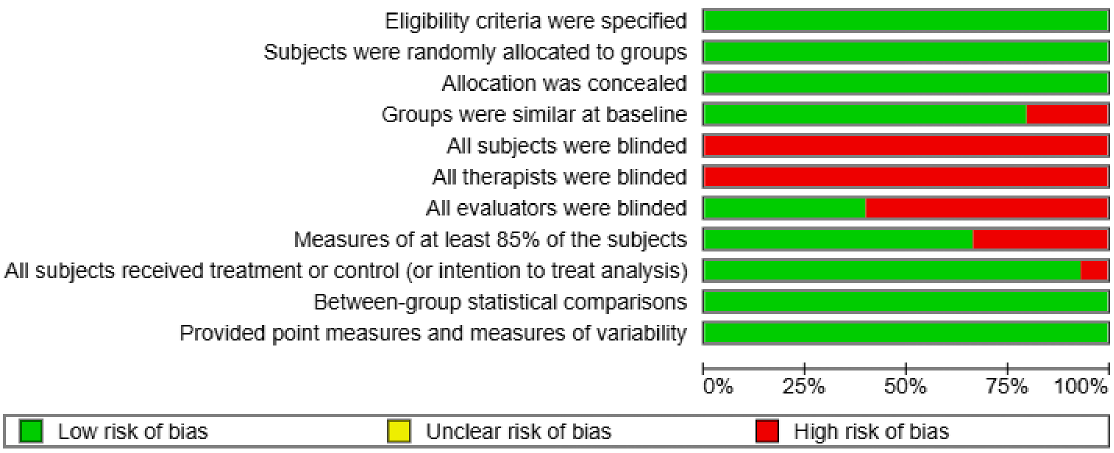

Figure S3. Forest plot of PNN50.

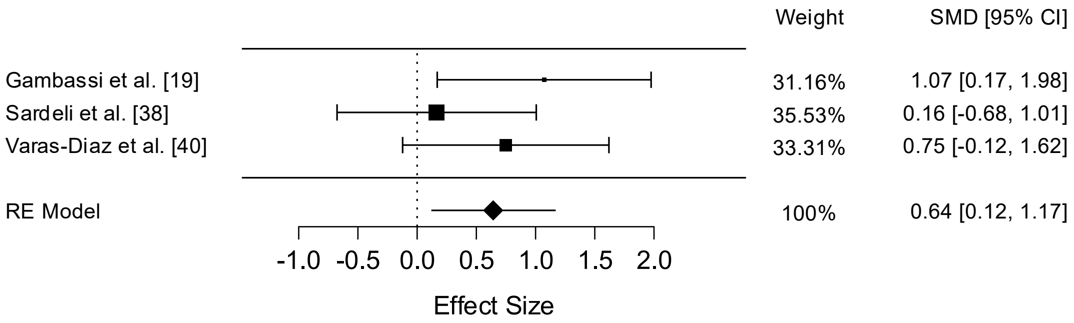

Figure S4. Forest plot of SD1.

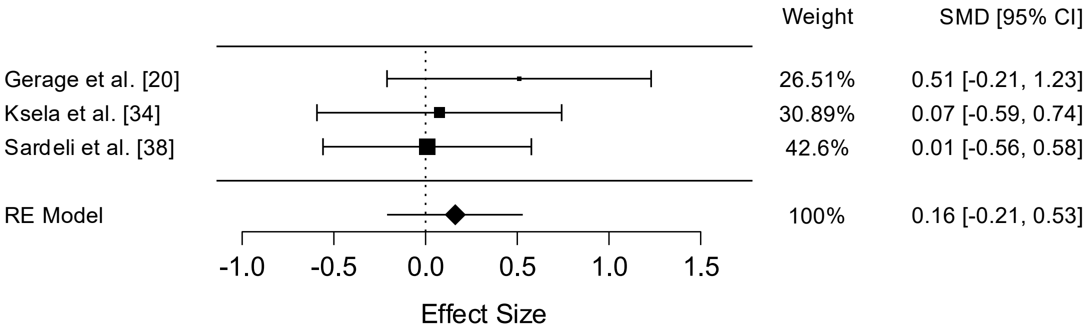

Figure S5. Forest plot of SD2.

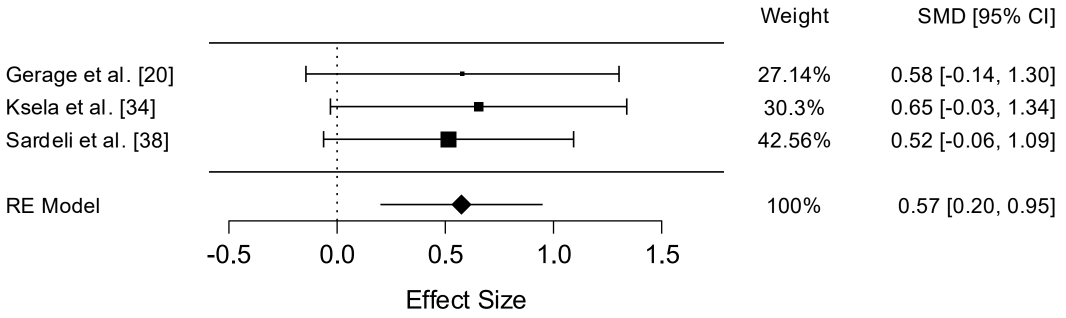

Figure S6. Forest plot of baroreflex sensitivity (BRS).

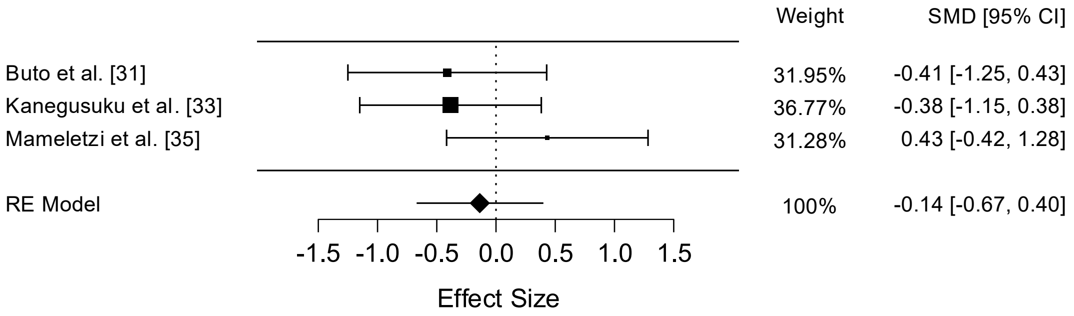

Supplement: Supplementary file 1 — Supplementary file1 (PDF 239 KB) [file 40279_2025_2357_MOESM1_ESM.pdf]
